# Parents’ Fears about Hospital Visits and Trait Anxiety in the COVID-19 Pandemic †

**DOI:** 10.3390/healthcare11081080

**Published:** 2023-04-10

**Authors:** Atsuko Nakano, Akihiro Maeta, Yuri Takaoka, Keigo Saeki, Masaaki Hamada, Yukiko Hiraguchi, Tomoko Kawakami, Ikuo Okafuji, Yutaka Takemura, Kyoko Takahashi, Makoto Kameda

**Affiliations:** 1 Department of Pediatrics, Kokuho Central Hospital, Nara 6360302, Japan; 2 Department of Food Science and Nutrition, School of Food Science and Nutrition, Mukogawa Women’s University, Hyogo 6638558, Japan; 3 Department of Pediatrics, Osaka Prefectural Hospital Organization Osaka Habikino Medical Center, Osaka 5830872, Japan; 4 Department of Epidemiology, Nara Medical University School of Medicine, Nara 6348522, Japan; 5 Department of Pediatrics, Yao Municipal Hospital, Osaka 5810069, Japan; 6 Department of Pediatrics, Osaka Saiseikai Nakatsu Hospital, Osaka 5300012, Japan; 7 Department of Pediatrics, Sumitomo Hospital, Osaka 5300005, Japan; 8 Department of Pediatrics, Kobe City Hospital Organization Kobe City Medical Center General Hospital, Hyogo 6500047, Japan; 9 Department of Pediatrics, Kindai University Hospital, Osaka 5898511, Japan

**Keywords:** allergy and immunology, anxiety, COVID-19, pediatrics, surveys and questionnaires

## Abstract

Anxiety in parents of children with allergic diseases during the COVID-19 pandemic may impact hospital visits. This study explored the effect of the pandemic on parents’ fears about hospital visits and their relationship with their personality traits. A cross-sectional, questionnaire-based study was conducted between September 2020 and March 2021, with parents of children aged 0–15 years, who regularly visited 24 outpatient facilities for allergic disease. The survey included patient information, fears about hospital visits, desired information, and the State-Trait Anxiety Inventory. Responses were compared between parents with high and low trait anxiety. The response rate was 97.6% (2439/2500). The most common fear was “Fear of getting medical care as usual (85.2%)” and “Fear of COVID-19 infection during hospital visits (87.1%)”. High trait anxiety showed a significant association with “Fear of worsening of children’s allergies” (adjusted OR: 1.31, 95%CI: 1.04 to 1.65, *p* = 0.022), and “Fear of worsening of COVID-19 due to allergy” (adjusted OR: 1.52, 95%CI: 1.27 to 1.80, *p* < 0.01). Healthcare professionals should share updates on COVID-19 and healthcare system to reduce parents’ fear. Subsequently, they should communicate the importance of continuing treatment to prevent worsening of COVID-19 and avoid emergency visits, considering parental trait anxiety.

## 1. Introduction

The rapid expansion of the novel coronavirus disease 2019 (COVID-19) [1] has had a significant impact on the treatment of allergy. After the World Health Organization declared COVID-19 as a global pandemic, the state of emergency in Japan was expanded to all prefectures on April 16, 2020. Japanese people were asked to reduce interaction with other people by 70–80% and avoid unnecessary outings for 1–2 months [2]. In addition, educational institutions were closed by the Government of Japan from March to May, 2020 [3].

The COVID-19 pandemic had a significant and widespread impact on the healthcare system. Parents of children with allergic diseases require not only daily care, but also emergency outpatient visits for the treatment of unexpected allergic reactions. Therefore, the chaos in the healthcare system caused by the COVID-19 pandemic may increase their burden. Furthermore, due to individual differences in the general disposition of anxiety, the perceived parental anxiety caused by the chaos in the healthcare system during such situations may vary.

Regarding the visit to medical institutions during the COVID-19 pandemic, there have been multiple reports on the relationship between patients’ anxiety and consultation frequency [4,5]. However, there is no report on the relationship between the degree of trait anxiety and consultation behavior during the pandemic. We hypothesized that the impact of parents’ fears about hospital visits during a pandemic could be more pronounced in those with high trait anxiety.

The purpose of the present study was to elucidate the prevalence of parents’ fears about hospital visits during the COVID-19 pandemic and investigate the association between their fears and personality traits. It can also be inferred that the results of the study can also apply to other categories of patients who require constant or periodic medical surveillance such as oncology patients, pregnant women, heart-failure patients, etc.

## 2. Materials and Methods

### 2.1. Study Design and Participants

This was a cross-sectional questionnaire-based study conducted in 24 facilities (including allergy hospitals, regional central hospitals, and two other clinics), staffed by physicians who specialized in pediatric allergic diseases, in Osaka, Hyogo, and Nara prefectures. Parents of children aged 0–15 years who often frequented the outpatient department for allergic disease were included. All parents provided informed consent to participate in the anonymous survey. Most parents completed the questionnaire at the hospital. Responses from those who were not the parents of regular pediatric outpatients with allergic disease, responses from non-parents, and responses with missing data on the relationship of the respondent with these children were excluded.

This study was performed from September 2020 to March 2021 with the approval of the ethics committees of the participating hospitals and Kokuho Central Hospital (approval number: 20-113).

### 2.2. Questionnaire

The questionnaire consisted of four parts. The survey items included questions on basic patient information, fears about hospital visits during the pandemic, information desired by parents, and the State-Trait Anxiety Inventory (STAI) [6].

#### 2.2.1. Basic Patient Information

We inquired about the relationship of the respondent to the pediatric patient, the age and sex of the children, prevalence of allergic disease and anaphylaxis, and whether they had adrenaline for self-injection (EpiPen).

#### 2.2.2. Parents’ Fears about Hospital Visits

We asked fears about hospital visits using the following: “Q1: Did you experience any anxiety as to whether you would be able to have normal consultations at the hospital? (Fear of getting medical care as usual)”, “Q2: Did you worry that by going to the hospital, your family could become infected with COVID-19? (Fear of COVID-19 infection during hospital visits)”, “Q3: Did you ever think that if your children’s allergies became slightly worse compared to before the spread of COVID-19, that ‘it can’t be helped’? (Fear of worsening of children’s allergies)”, “Q4: Have you ever worried about the opinions of others and thought about postponing or canceling a scheduled outpatient appointment/test/inpatient treatment? (Cancellation of a scheduled medical care)”, “Q5: Have you ever experienced anxiety that due to allergies, a COVID-19 infection could become worse? (Fear of worsening of COVID-19 due to allergy)”, and “Q6: Have you ever experienced anxiety in respect to allergy treatment not progressing as expected, when compared to before the spread of COVID-19? (Fear of delay in treatment of children’s allergies)”. These items were rated on a 5-point scale (“Quite a lot”, “A lot”, “Some”, “Not really”, and “None”).

#### 2.2.3. Information Desired by Parents

The respondents selected a maximum of three from the eight items (desired information: DI 1–8) as desired information from medical institutions. The items were as follows: “The effect of allergic conditions on COVID-19 infection (DI 1)”, “Information about infection prevention measures and infection risk at the hospital of attendance (DI 2)”, “What to do when experiencing problems with allergy treatment during the pandemic (DI 3)”, “What to do about outpatient examinations and hospitalization for testing (tolerance tests, etc.) during the pandemic (DI 4)”, “How to get an emergency examination during the pandemic (DI 5)”, “How to continue medications and/or treatments at home during the pandemic (DI 6)”, “How to utilize phone/remote consultation services during the pandemic (DI 7)”, and “Other (DI 8)”.

#### 2.2.4. State–Trait Anxiety Inventory (STAI)

The State–Trait Anxiety Inventory (STAI) is the most widely used questionnaire to assess two aspects of anxiety: it is used to measure the degree of anxiety in patients with more than 60 cultural and linguistic adaptations and a variety of physical and psychological disorders. The two aspects of anxiety are “state anxiety”, which is a temporary anxiety that occurs in certain situations, and “trait anxiety”, which refers to a personality’s susceptibility to anxiety. We were particularly interested in trait anxiety. Based on their scores of Q21 to Q40 in STAI, the parents were divided into two groups: the high-trait anxiety group (the high group; Japanese men with ≥44 points and Japanese women with ≥45 points) and the low-trait anxiety group (the low group; Japanese men with <44 points and Japanese women with <45 points) [6].

### 2.3. Statistical Analysis

We aggregated and analyzed responses to the questionnaire about parents’ anxiety regarding the hospital visits, relationship between the fears about hospital visits and parents’ trait anxiety, and information that parents wanted to receive from the hospital to continue treatment. Regarding the fears about hospital visits, the responses of “Quite a lot”, “A lot”, and “Some” were classified as “prevalent fear”, whereas “Not really” and “None” were classified as “no fear”. We compared the prevalence of the six kinds of fears between the high and low trait anxiety groups. We investigated the association between trait anxiety and fears about hospital visits using a multivariable logistic regression model, which included confounding factors, such as gender of parents, history of anaphylaxis, and possession of EpiPen. A history of anaphylaxis or possession of EpiPen is likely to indicate a severe case of allergic disease. Because severity of illness affects anxiety, we chose these as confounders. *p* < 0.05 was considered statistically significant. All statistical analyses were performed using EZR version 1.54 (Saitama Medical Center, Jichi Medical University, Saitama, Japan).

### 2.4. Source of Research Funding and the Role of the Sponsor

This study was supported by a Grant-in-Aid for Scientific Research by the Ministry of Education, Culture, Sports, Science, and Technology, Japan (Research project title: Development of a therapy-related psychological burden scale in oral immunotherapy for food allergy and relationship between stress and the outcome. Grant-in-aid for Young Scientists (2019–2022)), https://kaken.nii.ac.jp/ja/grant/KAKENHI-PROJECT-19K14042/ (accessed on 6 April 2023).

## 3. Results

**Figure 1:**
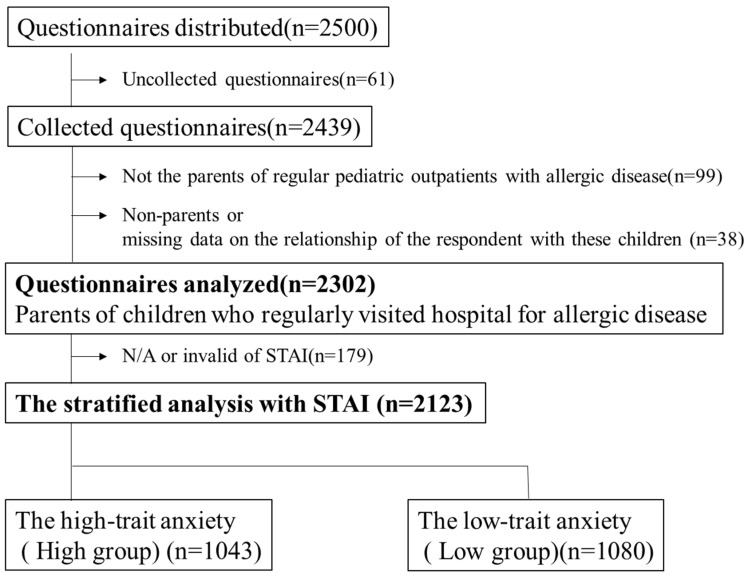
Flowchart of the participant selection process.

A total of 2500 questionnaires were distributed, and 2439 responses of the questionnaire were collected (response rate: 97.6%). Responses from individuals who did not regularly visit for allergic disease (n = 99), non-parents (n = 5), and questionnaires with missing data on the relationship with the children (n = 33) were excluded from the analysis. The parents who regularly visited the hospital for children’s’ allergic diseases (n = 2302) were included in the analysis. Parents with valid responses to the STAI trait anxiety items were included in the stratified analysis (n = 2123) (Figure 1). Of the 2302 respondent parents, 92.5% were answered by mothers. The median age of the children (n = 2451) was 3.8 years, the prevalence of food allergy, bronchial asthma, and history of anaphylaxis were 72.3%, 31.5%, and 12.4%, respectively (Table 1).

The fear of contracting a COVID-19 infection during hospital visits (Q2) was the most prevalent (87.1%), followed by the fear of getting medical care as usual (Q1, 85.2%), the fear of worsening of COVID-19 due to allergy (Q5, 54.6%), cancellation of scheduled medical care (Q4, 50.8%), the fear of delay in treatment of children’s allergies (Q6, 24.4%), and the fear of worsening of children’s allergies (Q3, 17.3%) (Table 2).

In univariable analysis, high trait anxiety was significantly associated with fear of worsening of children’s allergies (Q3; OR: 1.26, 95%CI: 1.04 to 1.53, *p* = 0.02), fear of worsening of COVID-19 due to allergy (Q5; OR: 1.20, 95%CI: 1.11 to 1.30, *p* < 0.01), and fear of delay in treatment of children’s allergies (Q6; OR: 1.71, 95%CI: 1.46 to 2.01, *p* < 0.01). After adjustment for potential confounders, high trait anxiety showed a significant association with fear of worsening of children’s allergies (Q3; adjusted OR: 1.31, 95%CI: 1.04 to 1.65, *p* = 0.022), and fear of worsening of COVID-19 due to allergy (Q5; adjusted OR: 1.52, 95%CI: 1.27 to 1.80, *p* < 0.01) (Table 3).

The information most respondents requested was “The effect of allergic conditions on COVID-19 infection” (DI 1, 23.2%). This was followed by “How to get an emergency examination during the pandemic” (DI 5, 19.5%) and “Information about infection prevention measures and infection risk at the hospital of attendance” (DI 2, 17.5%) (Table 4).

## 4. Discussion

The most common fear among parents was “Fear of getting medical care as usual” (Q1) and “Fear of COVID-19 infection during hospital visits” (Q2). High response rate of the questionnaire (97.6%) of this multi-center study suggests the generalizability of its evidence. To the best of our knowledge, this is the first study revealing that trait anxiety of parents of patients with allergy is significantly associated with 52% higher odds for fear of worsening of COVID-19 due to allergy and 31% higher odds for fear of worsening of children’s allergies independent of the gender of parents, history of anaphylaxis of patients, and prescription of EpiPen.

In the present study, 85.2% of the parents reported fear of getting medical care as usual (Q1). This result is consistent with findings that many parents required information on “How to deal with unexpected symptoms of allergies (DI 3)” and “How to get an emergency consultation (DI5)”. For parents of patients with allergy, whether they could receive medical care as usual is a major concern because the children have the possibility of emergency visits due to unexpected severe symptoms induced by an allergy. During the pandemic, many sick people around the world were not able to receive regular medical care [7,8,9]. However, regarding the medical care provision system, not all medical institutions interrupted their regular medical care during the survey period in Japan. Many facilities were able to maintain the regular medical care provision system. The fears of hospital visits could be alleviated by communicating the actual situation.

Most parents reported the fear of COVID-19 infection during hospital visits (Q2). This finding reflects the desire to avoid visiting a medical institution to avoid the risk of infection. This is consistent with the previous evidence that there was remarkable refraining from seeing a doctor during the pandemic [5,10,11,12,13]. Celik et al. reported that parents of patients with epilepsy with a risk of unscheduled emergency visits were more anxious about COVID-19 infection associated with hospital visits than parents of patients without it [14]. Similarly, parents of patients with allergy may be more sensitive than those of healthy children due to the possibility of unscheduled emergency visits. Sufficient information may reduce the fears of parents. For example, many facilities in Japan implement infection control by providing, for example, treatment for allergic reactions and COVID-19 at different times and places. Multiple societies, including the Japan Pediatric Society, have provided guidelines to protect both patients and healthcare professionals [15,16,17].

Healthcare professionals may support parents by sharing daily updates on the disease characteristics of COVID-19, the healthcare system, etc., to reduce excessive fear in parents so that their children can receive the standard treatment for allergic reactions [11,12].

Trait anxiety of parents is associated with “Fear of worsening of children’s allergies (Q3)” and “Fear of worsening of COVID-19 due to allergy (Q5)”. Papadopoulos et al. reported that parents of pediatric patients with food allergy and atopic dermatitis had higher anxiety and stress than parents of those without underlying illness [18,19]. However, it was unclear whether state anxiety or trait anxiety were associated with allergic symptoms. In the present study, we quantified the state anxiety and trait anxiety using the STAI and investigated the relationship with fear of medical treatment. Due to the cross-sectional design of the present study, we could not determine the causal direction between them. Trait anxiety may cause fear of medical treatment. In contrast, long-term allergic care for children may affect parents’ trait anxiety. Trait anxiety of parents of patients with allergy may be increased due to years of exposure to daily risks, such as the risk of unexpected symptoms from allergen intake for patients allergic to food and the risk of wheezing when catching a cold for patients with bronchial asthma. In other words, long-term allergic care may increase parental trait anxiety and make them sensitive to fears of allergic disorders. Increased trait anxiety depends on several factors, for example, the character traits of the parents with respect to anxiety, the severity of the acute illness, and the quality of medical guidance received from physicians in normal and special settings (pandemics) regarding the management of the child’s illness.

Regarding fear of worsening of children’s allergies, it can be inferred that parents prioritize COVID-19 measures over allergy measures during the pandemic. However, proper management of allergies makes it difficult for COVID-19 to become more serious, and allergy management reduces the risk of emergency hospital visits and also prevents COVID-19 infection by hospital visits. In addition, there are reports that allergy control has improved due to increased infection control and care time during the pandemic [19]. Daily allergy care is important even during the pandemic. In addition, there is a report that the management status of chronic diseases can be improved even in a pandemic by using telemedicine [20]. It is important to inform people of the advantages of self-restraint in daily life and the advantages of telemedicine.

Regarding fear of worsening of COVID-19 due to allergy, it was generally known that people with underlying diseases such as heart disease and diabetes had a high risk of COVID-19 aggravation from the early stages of the epidemic [21]. It has been clarified that the disadvantages peculiar to allergic diseases are small, and that well-managed asthma does not increase the severity of COVID-19 [15,22,23,24], but rather that it is less likely to become severe in allergic diseases. Communicating this information was thought to be effective in reducing anxiety among parents.

This study has some limitations. First, the participants in this study were parents who accompanied their children for the treatment during the pandemic. Because parents who did not visit the hospital were not included in this study, the actual proportion of parents with high trait anxiety may be higher. Second, because this was an anonymous survey, the medical records of patients were not linked to their responses to the questionnaire. Third, the cumulative number of COVID-19 infections was lower in Japan than in other developed countries such as the United States and European countries. Therefore, it was suggested that the impact of the COVID-19 pandemic may be smaller than in other developed countries.

## 5. Conclusions

We clarified the common fears among parents of patients with allergy and revealed the independent association between trait anxiety and the fears of medical treatment. Healthcare professionals may be able to support parents by sharing updates on COVID-19 and the healthcare system to reduce excessive fear in parents so that their children can receive the standard treatment. Moreover, they need to communicate the importance of continuing treatment to prevent worsening of COVID-19 and avoid emergency visits, taking into account parental trait anxiety. The results of this survey, which focuses on trait anxiety, will also be applied to other categories of patients who require constant or regular medical surveillance, such as oncology patients, pregnant women, and patients with heart failure. This is a good opportunity to examine the impact of the pandemic burden of COVID-19.

## Figures and Tables

**Table 1:** Based information of parents and children ^1^.

	Number (%) or Median (25th–75th Percentile)
Parents	2302
Relationship	
Father	172 (7.5%)
Mother	2130 (92.5%)
State Trait Anxiety Inventory (STAI) of parents (n = 2123)	
Angor (anxiety as a now state)	46.5 (40–53)
Anxietas (anxiety as personality trait)	45.4 (38–52)
Children	2451
Age (year) (n = 2451)	3.8 (3–9)
Sex (n = 2451)	
Boys	1527 (62.3%)
Girls	905 (36.9%)
N/A	19 (0.8%)
Prevalence of allergic diseases, multiple answers (n = 2451)	
Food allergy	1771 (72.3%)
Bronchial asthma	773 (31.5%)
Atopic dermatitis	1034 (42.2%)
Allergic rhinitis	707 (28.8%)
Allergic conjunctivitis	104 (4.2%)
History of anaphylaxis (n = 2451)	
Yes	304 (12.4%)
No	2147 (87.6%)
Prescription of adrenaline for self-injection (EpiPen) (n = 2451)	
Yes	669 (27.3%)
No	1782 (72.7%)

^1^ Data of age of children and STAI of parent were shown in the median and interquartile range. All other data are number and percentage. N/A: no answer.

**Table 2:** Prevalence of fears among 2302 parents.

Kinds of Fears		Prevalence, Number (%) ^1^ (n = 2303)
Q 1: Fear of getting medical care as usual	Fear (“Quite a lot”, “A lot”, and “Some”)	1962 (85.2%)
	No (“Not really”, and “None”)	341 (14.8%)
Q 2: Fear of COVID-19 infection during hospital visits	Fear (“Quite a lot”, “A lot”, and “Some”)	2006 (87.1%)
	No (“Not really”, and “None”)	297 (12.9%)
Q 3: Fear of worsening of children’s allergies	Fear (“Quite a lot”, “A lot”, and “Some”)	398 (17.3%)
	No (“Not really”, and “None”)	1905 (82.7%)
Q 4: Cancellation of scheduled medical care	Fear (“Quite a lot”, “A lot”, and “Some”)	1170 (50.8%)
	No (“Not really”, and “None”)	1133 (49.2%)
Q 5: Fear of worsening of COVID-19 due to allergy	Fear (“Quite a lot”, “A lot”, and “Some”)	1257 (54.6%)
	No (“Not really”, and “None”)	1046 (45.4%)
Q 6: Fear of delay in treatment of children’s allergies	Fear (“Quite a lot”, “A lot”, and “Some”)	561 (24.4%)
	No (“Not really”, and “None”)	1742 (75.6%)

^1^ These items were rated on a 5-point scale (“Quite a lot”, “A lot”, “Some”, “Not really”, and “None”). The responses of “Quite a lot”, “A lot”, and “Some” were classified as “prevalent fear”.

**Table 3:** Prevalence of fears by personality traits and odds ratio for fears.

Kinds of Fear	Prevalence, Number (%) ^1^			Odds Ratio (OR) for Fears			
		High Trait Anxiety ^2^ (n = 1043)	Low Trait Anxiety ^2^ (n = 1080)	Crude OR (95%CI)	*p* Value	Adjusted OR (95%CI) ^3^	*p* Value
Q 1: Fear of getting medical care as usual	Fear	891 (85.4%)	924 (85.6%)	1.00 (0.96–1.03)	0.90	0.97 (0.76–1.23)	0.79
	No	152 (14.6%)	156 (14.4%)				
Q 2: Fear of COVID-19 infection during hospital visits	Fear	911 (87.3%)	947 (87.7%)	1.00 (0.97–1.03)	0.86	0.96 (0.74–1.24)	0.75
	No	132 (12.7%)	133 (12.3%)				
Q 3: Fear of worsening of children’s allergies	Fear	196 (18.8%)	161 (14.9%)	1.26 (1.04–1.53)	0.02	1.31 (1.04–1.65)	0.02
	No	847 (81.2%)	919 (85.1%)				
Q 4: Cancellation of scheduled medical care	Fear	537 (51.5%)	541 (50.1%)	1.03 (0.95–1.12)	0.54	1.05 (0.88–1.24)	0.59
	No	506 (48.5%)	539 (49.9%)				
Q 5: Fear of worsening of COVID-19 due to allergy	Fear	624 (59.8%)	536 (49.6%)	1.20 (1.11–1.30)	<0.01	1.52 (1.27–1.80)	<0.01
	No	419 (40.2%)	544 (50.4%)				
Q 6: Fear of delay in treatment of children’s allergies	Fear	316 (30.3%)	191 (17.7%)	1.71 (1.46–2.01)	<0.01	0.97 (0.76–1.23)	0.79
	No	727 (69.7%)	889 (82.3%)				

^1^ These items were rated on a 5-point scale (“Quite a lot”, “A lot”, “Some”, “Not really”, and “None”). The responses of “Quite a lot”, “A lot”, and “Some” were classified as “prevalent fear”, ^2^ Based on their scores of Q21 to Q40 in STAI, the parents were divided into two groups: the high-trait anxiety group (the high group; men with ≥44 points and women with ≥45 points) and the low-trait anxiety group (the low group; men with <44 points and women with <45 points) [6]. ^3^ Adjusted for gender of respondents, history of anaphylaxis, and prescription of adrenaline for self-injection (EpiPen) CI: Confidence interval.

**Table 4:** Prevalence of fears among 2302 parents ^1^.

Items (Respondents Selected up to Three out of Eight Items)	Number (%)
DI 1: The effect of allergic conditions on COVID-19 infection	1377 (23.2%)
DI 2: Information about infection prevention measures and infection risk at the hospital of attendance	1041 (17.5%)
DI 3: What to do when experiencing problems with allergy treatment during the pandemic	987 (16.6%)
DI 4: What to do about outpatient examinations and hospitalization for testing (tolerance tests, etc.) during the pandemic	576 (9.7%)
DI 5: How to get an emergency examination during the pandemic	1149 (19.5%)
DI 6: How to continue medications and/or treatments at home during the pandemic	342 (5.8%)
DI 7: How to utilize phone/remote consultation services during the pandemic	441 (7.4%)
DI 8: Other	20 (0.3%)

^1^ Data are presented as number and percentage for all answers. The respondents selected up to three out of eight items as desired information from medical institutions.

## Data Availability

Data available on request due to restrictions eg privacy or ethical.

## Appendix A. List of Primary Investigators and Co-Investigators

Primary Investigators

Akagawa Shohei MD, PhD ^1^, Anzai Kaori, MD ^2^, Bandou Kenji, MD ^3^, Doi Masaaki, MD, PhD ^4^, Enomoto Masahiro, MD, PhD ^5^, Fujikawa Shiori, MD ^6^, Ikeda Akiko, MD ^7^, Nagai Megumi MD, PhD ^8^, Nishiyama Atsuko, MD ^9^, Otsuka Keita, MD ^10^, Shimizu Satoko, MD^11,12^, Sugimoto Yukiko, MD ^13^, Sumimoto Shinichi, MD, PhD ^2^, Tanaka Yukiko, MD ^14^, Tanaka Yuko, MD ^15^, Tanaka Yuya, MD ^16^, Wakahara Ryohei, MD, PhD ^17^, Yamasaki Koji, MD, PhD ^18^.

Co-Investigators

Arima Tomoyuki, MD ^8^, Fukasawa Yohei, MD ^21^, Hashimoto Naoki, MD, PhD ^19^, Kim Jong Soo, MD ^20^, Imaide Aya, MD ^6^, Masumi Hiroki MD ^8^, Matsutani Eri, MD ^3^, Momo Natsuki MD ^16^,Nakai Yoko, MD ^1^, Nakamichi Erina, MD ^2^, Onaka Masayuki, MD ^9^, Shigekawa Amane MD ^21^, Shingaki Tomoya, MD ^20^, Sunaga Ayana, MD ^3^, Tsurinaga Yuki, MD ^21^, Ueno Rumi MD ^21^, Yamada Saki, MD ^20^, Yamagishi Mitsuru, MD ^1^, Yamaguchi Tomohiro MD ^21^, and Yoshida Yukinori, MD, PhD ^21^

1. Kansai Medical University Hospital
2. Japanese Osaka Red Cross Hospital
3. Izumi City General Hospital
4. Higashiosaka City Medical Center
5. Takatsuki General Hospital
6. Abeno Medical Clinic
7. Yamatotakada Municipal Hospital
8. Kindai University Hospital
9. Nara Prefecture General Medical Center
10. Nara City Hospital
11. Matsushita Memorial Hospital
12. Shimizu Family Clinic
13. Hoshigaoka Medical Center
14. Kobe City Medical Center West Hospital
15. Osaka Police Hospital
16. Hyogo Prefectural Kobe Children’s Hospital
17. PL Hospital
18. Kaizuka City Hospital
19. Kokuho Central Hospital
20. Osaka Saiseikai Nakatsu Hospital
21. Osaka Habikino Medical Center

## References

[1] World Health Organization WHO Coronavirus Disease (COVID-19) Dashboard. https://covid19.who.int.

[2] Prime Minister’s Office of Japan Prime Minister Abe Press Conference on New Coronavirus Infection Japanese. https://www.kantei.go.jp/jp/98_abe/statement/2020/0407kaiken.html.

[3] Prime Minister’s Office of Japan 16th COVID-19 Control Headquarters Japanese. https://www.kantei.go.jp/jp/98_abe/statement/2020/0229kaiken.html.

[4] Japan Pediatric Society Survey of the Effects of Novel Coronavirus Infection on the Departments of Pediatrics of Pediatric Hospitals (Summary of the Second Survey) Japanese. http://www.jpeds.or.jp/uploads/files/20210520_shaho_nijichosa_gaiyo.pdf.

[5] Lazzerini M., Barbi E., Apicella A., Marchetti F., Cardinale F., Trobia G. (2020). Delayed access or provision of care in Italy resulting from fear of COVID-19. Lancet Child Adolesc. Health.

[6] Nakazato K., Shimonaka Y. (1989). The Japanese state-trait anxiety inventory: Age and sex differences. Percept. Mot. Skills.

[7] Horie H., Sata N. (2021). Limitations on medical practice in the Department of Surgery Limitations on medical practice in Department of Surgery, Jichi Medical University Hospital under the declaration of state of emergency. J. Jpn. Assoc. Operating Room Technol..

[8] Neil S., Carter R., Jones R., Roland D., Bayes N., Tavaré A., Hughes J., Turner T., Chynoweth J., Tan C. (2021). Caring for a sick or injured child during the COVID-19 pandemic lockdown in 2020 in the UK: An online survey of parents’ experiences. Health Expect..

[9] Tan C.D., Lutgert E., Neill S., Carter R., Jones R.B., Chynoweth J., Borensztajn D.M., Lakhanpaul M., Moll H.A. (2021). Parents’ experiences with a sick or injured child during the COVID-19 lockdown: An online survey in the Netherlands. BMJ Open.

[10] Imai K., Sampei M., Aurelie P., Okubo Y., Sawada N., Hosozawa M. (2022). Psychosocial factors associated to refraining from seeking medical care for children during the state of emergency: National survey of children’s quality of life and health in Japan during the COVID-19 pandemic. J. Jpn. Pediatr. Soc..

[11] Raucci U., Musolino A.M., Lallo D.D., Piga S., Barbieri M.A., Pisani M., Rossi F.P., Reale A., Atti L.M., Villani A. (2021). Impact of the COVID-19 pandemic on the Emergency Department of a tertiary children’s hospital. Ital. J. Pediatr..

[12] Ozturk A.B., Baççıoğlu A., Soyer O., Civelek E., Şekerel B., Bavbek S. (2021). Change in allergy practice during the COVID-19 pandemic. Int. Arch. Allergy Immnol..

[13] Smith A.C., Thomas E., Snoswell C.L., Haydon H., Mehrotra A., Clemensen J., Caffery L. (2020). Telehealth for global emergencies: Implications for coronavirus disease 2019 (COVID-19). J. Telemed. Telecare.

[14] Celik H., Acikel S.B., Ozdemir F.M.A., Aksoy E., Oztoprak U., Cucu E., Kucur O., Ceylan N., Yuksel D. (2021). Evaluation of the anxiety level of mothers of children with epilepsy during the COVID-19 pandemic period. Eur. Neurol..

[15] GINA Guidance about COVID-19 and Asthma Updated 26 April 2021. https://ginasthma.org/wp-content/uploads/2021/04/21_04_26-GINA-COVID-19-and-asthma.pdf.

[16] Japan Pediatric Society Treatment Guidelines for Novel Coronavirus infection (COVID-19) in Pediatric Outpatient Care. Japanese. https://www.mhlw.go.jp/content/000712473.pdf.

[17] Chu D.K., Aki E.A., Duda S., Solo K., Yaacoub S., Schünemann H.J. (2020). Physical distancing, face masks, and eye protection to prevent person-to-person transmission of SARS-CoV-2 and COVID-19: A systematic review and meta-analysis. Lancet.

[18] Papadopoulos N.G., Custovic A., Deschildre A., Mathioudakis A.G., Phipatanakul D., Wong G. (2020). Impact of COVID-19 on pediatric asthma: Practice adjustments and disease burden. J. Allergy Clin. Immunol. Pract..

[19] Lau G.Y., Patel N., Umasunther T., Gore C., Warner J.O., Hanna H., Phillips K., Zaki A.M., Hodes M., Boyle R. (2014). Anxiety and stress in mothers of food-allergic children. Pediatr. Allergy Immunol..

[20] Liu N., Huang R., Baldacchino T., Sud A., Sud K., Khadra M., Kim J. (2020). Telehealth for noncritical patients with chronic diseases during the COVID-19 pandemic. J. Med. Internet Res..

[21] Gasmi A., Peana M., Pivina L., Srinath S., Benahmed A.G., Semenova Y., Menzel A., Dadar M., Bjorklund G. (2021). Interrelations between COVID-19 and other disorders. Clin. Immunol..

[22] Wang J.Y., Pawankar R., Tsai H.J., Wu L.S.H., Kuo W.S. (2021). COVID-19 and asthma, the good or the bad?. Allergy.

[23] Skevaki C., Karsonova A., Karaulov A., Xie M., Renz H. (2020). Asthma-associated risk for COVID-19 development. J. Allergy Clin. Immunol..

[24] Carli G., Cecchi L., Stebbing J., Parronchi P., Farsi A. (2021). Is asthma protective against COVID-19?. Allergy.

